# Medical Relief under the New Poor-Law

**Published:** 1838-10

**Authors:** 


### MEDICAL RELIEF UNDER THE NEW POOR-LAW.

In our Ninth Number (p. 310) we announced our intention of soon considering in detail the subject of medical relief under the New Poor-law; but we deemed it right to wait the result of the enquiry about to take place before a committee of the House of Commons, directed, among other matters, to this particular subject. That enquiry has taken place; and we have now before us, in the 44th, 45th, and 46th Reports of the Committee, the evidence of the medical witnesses on all the points that have been agitated relating to the medical attendance on the sick poor. The gentlemen examined before the committee were, Sir Astley Cooper, Drs. Kay, Webster, R. D. Thomson, Elliotson, Hall, and Messrs. Rumsey, Ceely, Rowe, Farr, Evans, and Toogood; and, as all these gentlemen, with the exception perhaps of Dr. Kay, the assistant poor-law commissioner, were called by the committee who kindly undertook the management of the enquiry on the part of the British Medical Association and Provincial Medical Association, it is fair to take their evidence as exhibiting all the grievances which the profession had to complain of, and as affording the best suggestions for their relief. To the gentlemen just referred to, and above all to Mr. Wakley, (who conducted the examination of the witnesses, with much ingenuity and good sense, and great moderation,) the profession and the poor are greatly indebted, for the improvements which, we doubt not, will flow from this enquiry. We are happy to find that the improvements which we stated in the notice referred to as being especially wanted,—viz. "the establishment of fixed salaries in place of the system of *tenders*, the subdivision of districts, and the allotment of them with a more constant regard to the residence of the medical men," are likely to be all carried into effect. These were, indeed, the principal improvements recommended by the witnesses examined; the only others of much consequence being the appointment of medical assessors or guardians in each Union, and of one medical commissioner at the general Board in London. It will be seen by the official Report, which we subjoin, that both these recommendations have been rejected by the committee; and it is probable they will not be adopted by the government. We confess we regret extremely that the suggestion respecting a medical commissioner is not likely to be attended to; as we think such an addition to the Board is calculated to be of great advantage, both to the profession and the poor; although we think it of inferior importance to the other changes recommended, or likely to be adopted. We have great doubts, however, as to the other proposition respecting medical guardians: we are by no means sure that the creation of such a local authority might not rather tend to ferment than to allay the heats and jealousies which, we regret to say, too often disturb the harmony of medical men in small communities.

### *Report of the Committee.* (*Extract.*)

On the subject of medical relief, your committee examined several gentlemen connected with that profession. Mr. Rumsey, Secretary of the Provincial Medical and Surgical Association, complains that the paupers have a difficulty in obtaining medical assistance; that the size of the districts is too large, and the number of medical officers too small. He objects to medical men being controlled by persons incapable of judging of their proficiency; to their appointment by public advertisement and tender rather than on previous services and character; and he says the medical men accept these appointments, however inadequately remunerated, from an apprehension that strangers will be introduced, and that thus they might be deprived, not merely of the care of the poor, but of a part of their private practice. He produced instances of the too great size of medical districts, and of a reduction in the number of medical attendants on the poor; he says that in the former state of the law the attendance was often bad and the pay inadequate, and a want of control was felt, but that the districts were more convenient. The system of appointment by tender did also prevail under the old system, but not to such an extent as under the new. He approves of the weekly returns made to the board of guardians; and he suggests, as the principal points on which an amendment is required, the appointment, the remuneration, and the supervision of the medical officers. He would prefer a separate parochial appointment. If that be not assented to, that at least the districts should be smaller; and he suggests twelve miles square as the extent of a district. With respect to remuneration, he is of opinion that where the payment is by a fixed salary, the cases attended are too numerous, and that too many families receive medical relief; and that, on the contrary, where the payment is per case, the number attended is too small. He agrees, therefore, with Dr. Kay in suggesting that a scale of remuneration, embracing both those modes, should be adopted. This recommendation in substance is, that a list of persons entitled to medical relief, including those who are in the receipt of out-door relief with others who may be deemed necessarily indigent, should be made out and given to the medical officer, and that all those who are included in the list should be entitled to medical relief without the necessity of obtaining any order from any authority, and that the cost of administering this branch of medical relief should be estimated at a certain sum per case, so as to afford an adequate remuneration. Mr. Rumsey proposes, and Dr. Kay acquiesces in the proposal, that the scale of remuneration for those paupers should be about 6s. 6d. or 6s. per case. Mr. Rumsey supposes that this would be about double the present amount of remuneration. With respect to the casual paupers who would not be included in the list of those always entitled to relief, Dr. Kay and Mr. Rumsey think the remuneration should be higher, and that they should be paid for per case; Mr. Rumsey suggests 10*s*. as a reasonable sum, and that midwifery should be paid for separately. Mr. Rumsey afterwards brought forward another scale of remuneration, but which, though calculated upon a different basis, would in his estimation produce nearly the same result. He thinks, generally, that these arrangements must be governed in some degree by local peculiarities and the general rate of medical charges.

For the supervision or control of all medical arrangements, Mr. Rumsey proposes that a medical commissioner should be appointed by the Crown, to act with the present Poor Law Commissioners, and to whom all matters connected with this subject should be referred; and that two medical assessors should be appointed in each union, one by the medical men resident in the union, and the other by the board of guardians, by whom the medical arrangements should be generally settled. In case of dispute, reference to be made to the medical commissioner, whose decision should be final. Mr. Rumsey thinks it very desirable that every encouragement should be given to vaccination. He objects to the medical clubs, where medical men are compelled to attend them. He thinks those arrangements between independent labourers and medical men should be free from any control by the guardians, and be entirely voluntary. He approves of independent medical clubs, and produces the plan of one established in the south of Buckinghamshire.

The other medical witnesses examined by your committee concur generally in the observations and recommendations of Mr. Rumsey. Dr. Webster, president of the British Medical Association, describes himself to have lost his situation at Dulwich because he would not belong to a medical club. He thinks no medical district in a town should comprise a population of more than 10,000. Mr. Ceeley, surgeon of the Aylesbury Infirmary, complains of the introduction of medical men, strangers to that neighbourhood, in consequence of the proposals of the board of guardians not being accepted by the resident practitioners. He says that this was done under the influence of the Assistant Poor Law Commissioner. He admits the evils of the former system, and that there is now more employment for the agricultural labourers, whom he describes to be a sickly race of people, and suffering from insufficient food, especially since the introduction of the new law. The wages in one part of the Union of Aylesbury are not more than 6s. a week. He thinks it of great advantage that the same medical men who attend the rich should also have the care of the poor. He agrees with Mr. Rumsey as to the size of the districts, as to the appointment of a commissioner and assessors, and generally in other particulars. The remuneration for regular paupers should, he thinks, be at the rate of 7s. or 8s. per case, and for casual poor 12s.; fractures and dislocations to form a separate item. He estimates the amount of a reasonable remuneration at from four to seven per cent. on the total expenditure for the poor. He apprehends the danger of drugs of an inferior quality being administered, from inadequate salaries, and advises that a medicine chest containing the medicines most usually required should be placed in every parish to which the medical man could have immediate access, the expense of which he supposes might be from 3*l.* to 5*l.* for a parish of five hundred inhabitants.

Dr. Thomson gave evidence on the adulteration extensively carried on in drugs, and on the danger to which the poor especially may be exposed from the circumstance. Mr. Toogood, a surgeon of great experience from Bridgewater, agreed in the suggestions of the preceding witnesses. He thought that a medical district ought not to be above four or five miles in diameter. His private practice, however, generally extends to a distance of seven or eight miles. Dr. Elliotson and Dr. Marshall Hall express their general concurrence with the other witnesses; and Dr. Hall suggests that the medical officers, besides being of two years' standing in their profession, should have practised for one year at least within the union, or in its neighbourhood.

Mr. Farr, member of the council of the Medical Association, has examined with great care and labour the returns connected with this branch of the subject, which were ordered by the House. His evidence contains many valuable statistical details, and among other results of his enquires he states, that the average area of the present medical districts is about twenty-one square miles; that of the district of Newbury, which is co-extensive with the union, is seventy-two square miles. In many other instances the size of medical districts appears to him to be inconveniently large, where the extreme points are eleven or twelve miles from the residence of the medical officer. In some unions, where the salaries are fixed, sixty-eight persons in one thousand seem to be attended; where the payment is per case, the proportion attended is fifty in one thousand. The importance of medical arrangements for the treatment of the poor may be inferred from the fact, that nearly one-fifth of the deaths in this country take place under the care of the parochial medical officers. Mr. Farr alludes to the superior pay of medical attendance on gaols; and as the present remuneration of the parish surgeon is fixed at about 3s. 3d. per case, he would propose to double it; in all other respects he coincides with the recommendations which had been previously made to your committee.

Sir Astley Cooper, having had an opportunity of seeing the evidence which had been given by the witnesses already referred to, was of opinion that the present medical districts were too large. He objected to the appointment by tender; he thought that it might be advantageous that medical chests containing the usual and most easily compounded medicines should be kept in each parish; and he concluded by stating, that you should choose the same men to attend the poor as you would be satisfied with in your own family; and that the difficulties connected with this subject would be easily settled if the districts were made smaller, suggesting a diameter of about five miles, and if no person were appointed to a medical district who had not passed the examination of Apothecaries' Hall, and of a Board of Midwifery, and received a diploma from the College of Surgeons.

Your committee again examined Dr. Kay; and they directed the evidence which he gave at an earlier stage of their enquiry, upon the subject of the medical attendance of the poor, to be reprinted, in connexion with that of the other witnesses on this branch of the subject; most of the recommendations of the medical gentlemen who had been examined, as far as the administration and the remuneration of medical relief are concerned, were founded on suggestions originally made by Dr. Kay. In his subsequent examination, Dr. Kay advises that separate arrangements should be made for attendance on the workhouse, for which drugs should be provided by the guardians, under the direction of the Poor Law Commissioners, and a few of the surgical instruments in most frequent use. The scale of remuneration for the list of paupers before adverted to should, he thinks, be about 6s. per case, independent of the workhouse. He approves of the proposal with regard to parish medicine chests, but does not think the appointment of a medical commissioner or of medical assessors necessary. In all cases of difficulty, connected either with the general arrangements or with the medical treatment of the poor, recourse may be had, and is now had, to the advice of physicians, either by the board of guardians or by the Poor Law Commissioners.

On reviewing the whole of the evidence connected with medical relief, your committee are of opinion that while most of the witnesses called on this subject agree in representing many of the evils of the old system to have been corrected, yet it does appear that the size of the medical districts is in many instances inconveniently large, and that in some cases the poor have been assigned to the care of too small a number of medical officers. They think also that it may be desirable to discontinue the practice of advertising for tenders from the medical men; this seems to have given offence to a profession, whose feelings and wishes it is important to consult. The committee believe the practice to have been originally adopted experimentally, and merely with a view to ascertain what, in the opinion of those gentlemen, was a reasonable remuneration for their services; but if offers have been made and appointments accepted by the resident surgeons at a rate below a reasonable amount of remuneration, under an apprehension that strangers to the neighbourhood might be introduced, and that a part of their private practice might be lost, together with their attendance on the poor, your committee think that this is a circumstance to be regretted, and they advise the adoption of some different mode of appointment. A state of things may undoubtedly arise in which it may be impossible to come to fair terms with the resident medical men, and when the introduction of a stranger may be indispensable: in that case great care should be taken in the examination of the qualifications of an individual of whose practice the board of guardians could have had no previous experience. The suggestions on this point by a person of the authority and experience of Sir Astley Cooper are undoubtedly entitled to great weight. Your committee also feel it to be most important that the poor should be perfectly satisfied with their medical attendants; and with this view it appears to be desirable, as indeed is almost always the case, that the care of the poor should be confided to the same person who is in the habit of visiting their richer neighbours.

With respect to the amount of remuneration for medical services, your committee do not feel themselves able to give any opinion upon the precise sums which have been named; this question must in some degree be governed by local circumstances, by the number of practitioners, by the nature of the country, by the degree in which the residences of the poor are scattered or near together, and by the general rate of remuneration previously existing in the district. Whatever may be the amount of remuneration fixed in any union, your committee are of opinion that attendance on the sick should be made a parochial charge, each parish paying for its own cases; and that it should never be made a part of the establishment charge, and distributed among the different parishes in proportion to their averages. If it should be determined to adopt the scheme of making out, either at the beginning of the year, or at any other period, a list of the persons who shall be entitled to receive medical relief on their own application, one principal difficulty will consist in determining whose name shall be inserted in the list of the permanent poor. With those already in the receipt of out-door relief there will be no difficulty; but with respect to those who, when in health, can support themselves, but who may be supposed to be unable to meet the losses and expenses of sickness, it will require a very cautious discrimination in completing the list, by which the extent of medical relief to be given and the amount of the remuneration are mainly to be determined. Your committee, however, think that the principle on which those additional names are recommended for insertion is perfectly just; and, as they think that the board of guardians must decide on those, as on all individual cases, they have no reason to believe that their choice will be indiscreetly exercised; and they cannot avoid saying that while they would advise a proper caution to be used, they are of opinion with Mr. Gulson, and other witnesses, that medical relief may with great propriety be given more extensively than any other kind of parochial assistance.

Your committee are not disposed to concur in the suggestions which have been made for the appointment of a medical commissioner by the crown, and of medical assessors by boards of guardians and the resident medical practitioners. The same considerations which govern private individuals in the selection of medical attendants for themselves and their families will, it is hoped, influence boards of guardians in selecting attendants for the poor; the same individuals, in the great majority of instances, will attend the poor in common with the other inhabitants of the district; and in this respect no class of the population will be exposed to any comparative disadvantage; neither does it appear desirable that the appointment of medical officers should be taken from the boards of guardians, and revert to the several parishes of an union. Your committee, from a feeling of respect for the medical profession, and believing that their attendance on the poor has been marked by great liberality and humanity, are anxious that the suggestions which have been made by them should be favorably considered by those who are charged with the administration of the law. They recommend the evidence which they have received on this subject to the attention of the Poor Law Commissioners; and they cannot but hope that arrangements may be made to remove some of the objections reasonably entertained to the present practice, and to put this branch of relief on a footing which shall be satisfactory to the medical men and be conducive to the comfort of the poor.

It has been recommended, and your committee agree in the recommendation, that periodical reports to the boards of guardians as to the state of health prevalent in the medical districts should be required from the medical officers. It appears desirable that great care should also be exercised in requiring that the accounts of the diseases and treatment of the individuals attended should be accurately kept, and in subjecting those accounts to revision, as one means of security that the sick are carefully attended and correctly treated.

Upon the subject of medical relief the committee have agreed to the following resolution:

> That the administration of medical relief to the poor has been in many respects amended under the new law, but that there is still room for further improvement; that the medical districts, in some instances, seem to be inconveniently large; that they should be of such a size as to admit an easy access of the medical man to his patients; and that the remuneration should be such as to ensure proper attention and the best medicines.

